# Rational Matching of Metal–Organic Frameworks and Polymers in Mixed Matrix Membranes for Efficient Propylene/Propane Separation

**DOI:** 10.3390/polym16172545

**Published:** 2024-09-09

**Authors:** Zijun Yu, Yuxiu Sun, Zhengqing Zhang, Chenxu Geng, Zhihua Qiao

**Affiliations:** 1 School of Textile Science and Engineering, Tiangong University, Tianjin 300387, China; yuzijun@tiangong.edu.cn; 2 State Key Laboratory of Separation Membranes and Membrane Processes, Tianjin 300387, China; yuxiusun@tiangong.edu.cn (Y.S.); zhangzhengqing@tiangong.edu.cn (Z.Z.); 3 School of Chemical Engineering and Technology, Tiangong University, Tianjin 300387, China

**Keywords:** mixed-matrix membranes, metal–organic framework, improved compatibility, interface coordination, gas separation

## Abstract

The exploitation of high-performance membranes selective for propylene is important for developing energy-efficient propylene/propane (C_3_H_6_/C_3_H_8_) separation technologies. Although metal–organic frameworks with a molecular sieving property have been considered promising filler materials in mixed-matrix membranes (MMMs), their use in practical applications has been challenging due to a lack of interface compatibility. Herein, we adopted a surface coordination strategy that involved rationally utilizing carboxyl-functionalized PIM-1 (cPIM) and ZIF-8 to prepare a mixed-matrix membrane for efficient propylene/propane separation. The interfacial coordination between the polymer and the MOF improves their compatibility and eliminates the need for additional modification of the MOF, thereby maximizing the inherent screening performance of the MOF filler. Additionally, the utilization of porous PIM-1 guaranteed the high permeability of the MMMs. The obtained MMMs exhibited excellent separation performance. The 30 wt% ZIF-8/cPIM-1 membrane performed the best, exhibiting a high C_3_H_6_ permeability of 1023 Barrer with a moderate C_3_H_6_/C_3_H_8_ selectivity of 13.97 under 2 bars of pressure. This work presents a method that can feasibly be used for the preparation of defect-free MOF-based MMMs for specific gas separations.

## 1. Introduction

Light olefins, such as ethylene (C_2_H_4_) and propylene (C_3_H_6_), are essential energy resources and feed stocks for the production of important industrial chemicals and are mainly obtained via petrochemical processing [1,2,3]. Furthermore, high-purity olefins are in high demand, especially in polymer production factories [4,5,6]. Thus, the efficient separation of olefin/paraffin is an urgent need. However, the separation of olefins from paraffins is extremely challenging because of their similar molecular sizes and physical properties [7,8]. The traditional method of cryogenic distillation is a very energy-intensive strategy due to the phase transition process involved. In contrast, membrane technology is extremely efficient at separating light hydrocarbons and consumes less energy [9,10]. Membrane materials play a significant role in membrane technology, and thus a lot of effort has been devoted to developing different types of membrane materials and the exploitation of advanced membrane materials.

In the past few decades, polymers have been widely used as membrane materials in the realm of gas separation due to their excellent solution processability [11]. However, traditional polymer membranes often suffer from a trade-off between permeability and selectivity, which strongly impedes the further application of these polymer membranes in industrial separation [12]. To solve this problem, mixed-matrix membranes incorporating porous filler particles into continuously polymeric matrices have been widely explored for gas separation [13,14]. Among the numerous porous fillers available, metal–organic frameworks (MOFs) stand out due to the extraordinary advantages they have, such as a high surface area and pore volume, adjustable pore size, and chemical modifiability [15]. ZIF-8, as a representative MOF, shows outstanding C_3_H_6_/C_3_H_8_ separation potential because of its specific molecular-sieving apertures [16]. However, the mixed-matrix membranes that have been reported still have a separation performance marked by a trade-off between propylene/propane selectivity and propylene permeability, which may be attributed to their imperfect interfacial compatibility and the polymer matrices chosen. For example, Koros and coworkers fabricated a mixed-matrix membrane using 6FDA-DAM polyimide and ZIF-8, and the resulting optimal membrane, with 48.0 wt% filler loading, exhibited an ideal C_3_H_6_/C_3_H_8_ selectivity of 31.0, with a C_3_H_6_ permeability of 56.2 Barrer [17]. Ma et al. reported a mixed-matrix membrane composed of ZIF-8 and hydroxy-functionalized microporous polyimide PIM-6FDA-OH, which yielded a C_3_H_6_ permeability of 30 Barrer and a C_3_H_6_/C_3_H_8_ selectivity of ~31 [18]. Meanwhile, Jiang and coworkers reported a mixed-matrix membrane with ZIF-8 direct-through channels via the solution-casting approach, which exhibited a propylene permeability of 582 Barrer and a propylene/propane selectivity of 42.8 [19]. Therefore, rationally selecting a polymer matrix to match ZIF-8 and increasing their interfacial compatibility are key factors in developing high-performance C_3_H_6_/C_3_H_8_ separation membranes.

The interfacial compatibility between the filler and the polymer matrix in mixed-matrix membranes greatly impacts their gas separation performance. Insufficient interfacial compatibility can result in the creation of non-selective defects between the filler and the polymer, which impair the filler’s capacity to effectively sieve gasses. In contrast, strong interfacial compatibility enables the filler to fully utilize its gas sieving capabilities, thereby improving the selectivity and permeability of the membrane [8,9,10,11]. To improve the interfacial compatibility between MOF particles and their polymer matrix, various strategies have been proposed, which aim to boost the C_3_H_6_/C_3_H_8_ separation performance of the resulting MMMs [20,21]. In particular, Zou and coworkers reacted functionalized ZIF-8-CN with PIM-1 through covalent linkage under a thermal treatment and thus strengthened the interfacial compatibility of their resulting MMMs, which typically had a C_3_H_6_ permeability of ~370 Barrer and C_3_H_6_/C_3_H_8_ selectivity of ~28 [22]. Eddaoudi and coworkers reported a surface functionalization strategy used to improve the interfacial compatibility between KAUST-7 and polyimide, and the optimal membrane created displayed a C_3_H_6_ permeability of ~95 Barrer and a C_3_H_6_/C_3_H_8_ selectivity of ~20 [23]. Zhang and coworkers achieved a uniform dispersion of SIFSIX-3-Zn nanoparticles in PIM-1 by reducing the size of the MOF fillers, resulting in an optimal C_3_H_6_ permeability of 4102.1 Barrer with a C_3_H_6_/C_3_H_8_ selectivity of 7.9 [24]. Lee et al. achieved good MOF–polymer interface compatibility by using a defect engineering strategy in MOFs, and the prepared defect-engineered UiO-66/6FDA-DAM membrane exhibited an outstanding C_3_H_6_/C_3_H_8_ separation performance even under harsh operation conditions [25]. Additionally, in our previous work, we proposed a bilayer structure modification strategy for ZIF-67 to enhance its interface compatibility, with the resulting OLC-ZIF-67/XLPEO membrane displaying an enhanced C_3_H_6_ permeability (~266 Barrer) and a C_3_H_6_/C_3_H_8_ selectivity of 40.5 [26]. In addition to these wonderful works, a molecular-level interfacial interaction strategy was recently proposed for hybrid membranes with nearly defect-free interfaces, where MOF nanoparticles can be tightly wrapped within carboxylated polyimides through the strong coordination interaction or hydrogen bonding between MOF frameworks and –COOH groups [27,28,29].

**Figure 1:**
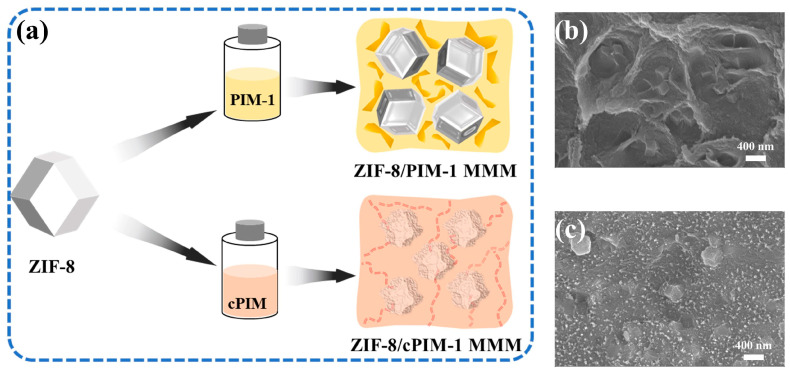
(**a**) Illustration of ZIF-8/PIM-1 and ZIF-8/cPIM membranes. Cross-sectional SEM images of ZIF-8/PIM-1 (**b**) and ZIF-8/cPIM (**c**) membranes at the same magnification, and with a ZIF-8 loading of 20 wt%, demonstrate their two distinct composite morphologies.

Inspired by this interfacial interaction strategy, we intentionally selected carboxyl-functionalized polymer of intrinsic microporosity-1 (cPIM) as our continuous polymer matrix and ZIF-8 as our filler material to form a hybrid membrane for efficient C_3_H_6_/C_3_H_8_ separation. cPIM was selected as the continuous matrix phase because of its porous nature and carboxylic acid groups. ZIF-8 is sensitive to acids, and the zinc sites on its surface could be exposed when encountering –COOH groups. Meanwhile, zinc ions can coordinate with –COOH and form a nearly defect-free interface. A diagram of the design concept is shown in Figure 1a. As expected, the obtained ZIF-8/cPIM membrane exhibited enhanced C_3_H_6_ permeability and C_3_H_6_/C_3_H_8_ selectivity in comparison to the ZIF-8/PIM-1 membrane under similar testing conditions. Moreover, the optimal ZIF-8/cPIM membrane, with 30% filler loading, showed long-term stability in its separation performance due to its improved interfacial compatibility.

## 2. Materials and Methods

### 2.1. Materials

5,5′,6,6′-Tetrahydroxy-3,3,3′,3′-tetramethyl-1,1-spirobiindene (TTSBI, 99%) and tetrafluoroterephthalonitrile (TFTBN, 99%) were purchased from Beijing Innochem Technology Co., Ltd. (Beijing, China). Potassium carbonate (K_2_CO_3_, 99%) was purchased from Anhui Energy Chemical Technology Co., Ltd. (Hefei, China). *N*,*N*-dimethylformamide (DMF), chloroform (CHCl_3_), and tetrahydrofuran (THF) were purchased from China National Pharmaceutical Reagent Co., Ltd. (Beijing, China). Acetic acid (CH_3_COOH, 98%) and concentrated sulfuric acid (H_2_SO_4_, 98%) were purchased from Tianjin Fengchuan Chemical Reagent Co., Ltd. (Tianjin, China). Propylene (C_3_H_6_), propane (C_3_H_8_), and helium (He) were supplied by Tianjin Taiya Gas Sales Co., Ltd. (Tianjin, China).

### 2.2. Synthesis of PIM-1 and cPIM

The synthesis of PIM-1 was based on a previous study [30]. Typically, TTSBI (10 g, 30 mmol) and TFTBN (6 g, 30 mmol) were dissolved in DMF (200 mL), followed by the dispersion of K_2_CO_3_ (10.5 g, 75 mmol) into the above solution. The mixture was then stirred at 65 °C in an N_2_ atmosphere for 72 h, then poured into 500 mL of methanol, washed three times with methanol to remove unreacted TTSBI and TFTBN, and stirred for 12 h in a 0.1 wt% hydrochloric acid aqueous solution to remove K_2_CO_3_. Yellow powder was obtained by filtration and dried in a vacuum oven at 120 °C for 12 h, collected, and sealed for future use.

The synthesis of cPIM refers to a previous report [31]. In brief, 130 mL of H_2_O, 130 mL of H_2_SO_4_, and 42 mL of CH_3_COOH were sequentially added to a 500 mL round-bottom flask. Two g of PIM-1 powder was weighed and dispersed into the above solution. The mixture was condensed and refluxed with stirring and heating at 150 °C for 3 h. After the reaction was completed, the reaction system was allowed to cool to room temperature. The mixture was filtered to obtain a dark yellow powder, which was subsequently heated in a weakly acidic aqueous solution at 120 °C for 12 h. Then, the mixture was filtered again to obtain cPIM powder. The resulting cPIM powder was dried in a vacuum oven at 120 °C for 12 h.

### 2.3. Synthesis of ZIF-8

The synthesis of ZIF-8 refers to a previous report [32]: ZnCl_2_ methanol solution (80 mL, 0.5 M) was added to Hmim methanol solution (300 mL, 3.5 M) and stirred for 3 h. After the reaction, the mixed solution was centrifuged, and the product was centrifuged 3 times with methanol. Finally, ZIF-8 powder was dried in a vacuum oven at 100 °C for 12 h.

### 2.4. Preparation of Membranes

The mixed-matrix membranes (MMMs) were prepared using the solution casting method, taking the preparation of ZIF-8/PIM-1 membrane as an example. A certain amount of ZIF-8 powder was first dispersed in 4 mL of chloroform solvent and sonicated for 60 min to achieve full dispersion, resulting in a uniformly dispersed suspension. Subsequently, a certain amount of PIM-1 powder was added to the aforementioned suspension and stirred at room temperature for 24 h. The mixture was ultrasonicated for 30 min to remove bubbles and obtain a uniform casting solution. Finally, the casting solution was poured into a polytetrafluoroethylene culture dish and allowed to evaporate naturally at room temperature for 24 h. The dry film was peeled off of the culture dish and placed in an 80 °C vacuum oven to thoroughly remove any residual solvent.

For the preparation of ZIF-8/cPIM membrane, a specific mass of ZIF-8 powder was first dispersed in 2 mL of THF solvent and sonicated for 60 min to achieve thorough dispersion, resulting in a uniformly dispersed suspension. Then, a specific mass of cPIM powder was dissolved in 2 mL of THF solution to obtain a homogeneous and transparent polymer solution. The polymer solution was subsequently added dropwise to the ZIF-8 suspension and stirred continuously at 0 °C for 3 h. Ultrasonication was carried out for 30 min to remove the air bubbles and to obtain a uniform casting solution. The subsequent steps were consistent with the preparation method for the ZIF-8/PIM-1 membrane.

The preparation for PIM-1 and cPIM membranes were performed as follows: First, a certain amount of PIM-1 and cPIM powders was dissolved in CHCl_3_ and THF solution, respectively, to obtain uniform and transparent polymer solutions. The subsequent steps were identical to the preparation method of ZIF-8/PIM-1 membranes. Additionally, the loading of MOF in the MMMs was calculated by the following Equation (1):

(1) $$ZIF-8Loading\left(wt\%\right)=\frac{m_{ZIF-8}}{m_{Polymer}+m_{ZIF-8}}$$

### 2.5. Characterization

The X-ray diffraction (XRD) spectra of ZIF-8 and membrane samples were obtained using a Bruker D2 Advance diffractometer in the range of 2θ for 2–50° with Cu target Kα radiation (λ = 1.54 Å) as the X-ray source. The BET (Brunauer Emmett Teller) specific surface area of ZIF-8 was measured by N_2_ adsorption using an ASAP 2020PLUS HD88 instrument at 77 K. The size and morphology of ZIF-8 particles, as well as the distribution of ZIF-8 within the membrane, were measured using SEM (Gemini 500). Attenuated total reflection Fourier transform infrared (ATR-FTIR) spectra in the range of 400–4000 cm^−1^ were obtained with the Bruker TENSOR II spectrophotometer. X-ray photoelectron spectroscopy (XPS) was performed on a Thermo Fisher K-alpha spectrometer.

### 2.6. Permeation Test

The gas separation performance was tested by homemade permeation equipment integrated with a gas chromatograph (Agilent 7890B). A mixture of C_3_H_6_/C_3_H_8_ (with a volume ratio of 50/50) was used as the feed gas and helium was used as the sweep gas, and the composition of the permeate gas was analyzed online using a gas chromatograph. The permeability of the component *i* in a mixed gas was calculated by the following Equation (2):

(2) $$P_{i}=\frac{Q_{i}l}{∆p_{i}A}$$

Among them, *P_i_* was the gas permeability (Barrer = 10^−10^ cm^3^ STP cm cm^−2^ s^−1^ cm Hg^−1^), *Q_i_* was the volumetric flow rate of gas *i* (cm^3^/s^−1^ (STP)), *l* was the membrane thickness (cm), *A* was the testing area of the membrane (cm^2^), and Δ*p_i_* was the pressure difference (cm Hg^−1^) between component *i* upstream and downstream of the membrane.

Selectivity α was calculated by the following Equation (3):

(3) $$\alpha_{i/j}=\frac{y_{i}/y_{j}}{x_{i}/x_{j}}$$

The solubility (*S*) and diffusivity (*D*) of the membrane for C_3_H_6_ and C_3_H_8_ were measured using a permeameter (G2/110-A, Jinan Languang Electromechanical Technology Co., Ltd., Jinan, China) based on the constant volume and variable pressure principle. The calculation formulas were as follows:

(4) $$D=\frac{l^{2}}{6\theta }$$

(5) $$P=\frac{Vl}{p_{0}ART}\frac{dp}{dt}\times 10^{10}$$

(6) $$S=\frac{P}{D}$$

Among them, *D* was the diffusivity of the membrane to gas (cm^2^/s), *l* was the thickness of the membrane (cm), *θ* was the time delay (s), *P* was the permeability coefficient of the membrane to gas (Barrer), *V* was the volume of the downstream chamber (cm^3^), *p_0_* was the upstream pressure (cmHg), *A* was the effective membrane area (cm^2^), *R* was the gas constant, *T* was the test temperature (K), and *S* was the solubility of the membrane to gas (cm^3^ STP cm^−3^ cm Hg^−1^).

## 3. Results

### 3.1. Characterization of ZIF-8 and MMMs

As shown in Figure S1, the synthesized ZIF-8 powder was characterized by XRD, BET, SEM, and ATR-FTIR analysis to investigate its chemical structures and porosities, and the results show that the synthesized sample was in good agreement with the simulated one, which proves the generation of the ZIF-8 phase [32]. In addition, the resulting ZIF-8 powder had a specific surface area of about 1600 m^2^ g^−1^, with a size of around 400 nm. The isothermal adsorption curves of C_3_H_6_ and C_3_H_8_ on the ZIF-8 particles at 298 K are shown in Figure S2, and are consistent with previously reported results [17,22]. Due to the excellent solubility of the polymer of intrinsic microporosity (PIM-1) and its accessible functionalization due to its cyano groups, the carboxylated PIM-1 (abbreviated as cPIM) was synthesized and used as a continuous matrix. The ATR-FTIR spectra of the cPIM exhibited distinct variations from those of pristine PIM-1, as shown in Figure S3. The emergence of a broad peak in the range of 3200–3730 cm^−1^ in the cPIM can be attributed to the hydroxyl (-OH) group. Moreover, the appearance of a distinct peak at approximately 1692 cm^−1^ corresponds to the carbonyl (C=O) stretching vibration, substantiating the successful introduction of carboxyl functionality into the cPIM framework [31,33]. These spectral features confirm the synthesis of cPIM with the targeted carboxyl modifications. Subsequently, as-synthesized ZIF-8 particles were incorporated into the PIM-1 and cPIM polymer matrices, respectively, to prepare corresponding MMMs. To facilitate a comparative evaluation of the dispersion and morphology of the ZIF-8 particles within these polymer matrices, SEM was employed to analyze the cross-sectional profiles of the two membrane configurations (Figure 1b,c). For the ZIF-8/PIM-1 membrane, ZIF-8 was homogeneously distributed within the PIM-1, and its morphology remained consistent with that of ZIF-8 particles. However, in the case of the ZIF-8/cPIM membrane, the surface of ZIF-8 within the membrane became significantly rougher and exhibited an encapsulated state. This phenomenon may be attributed to the coordination of the free carboxyl groups within the cPIM with the Zn sites in the ZIF-8 [34,35,36]. This effect was instrumental in augmenting the interfacial compatibility between the MOF and the polymer matrix, thereby enabling the preparation of highly loaded MOF-based MMMs.

**Figure 2:**
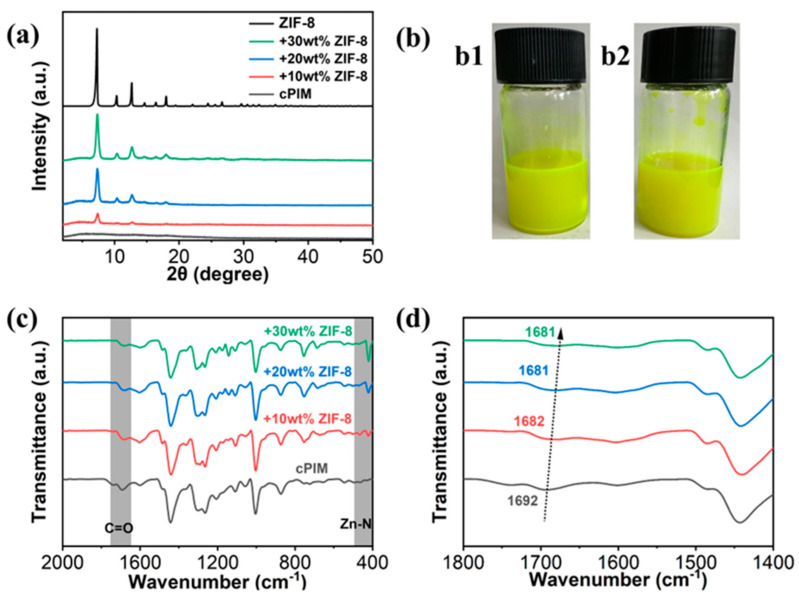
(**a**) XRD patterns of ZIF-8 nanoparticles and their associated MMMs with different filler loadings. (**b**) Optical images of cast ZIF-8/PIM-1 (**left**, precipitate) and ZIF-8/cPIM (**right**, homogeneous) membranes after being left to stand for 24 h, with a ZIF-8 loading of 20 wt%. (**c**,**d**) ATR-FTIR spectra and the according magnification part of cPIM and MMMs with different filler loading content.

The XRD patterns shown in Figure 2a and Figure S4a demonstrate that the ZIF-8 retained its structural integrity after its incorporation into the PIM-1 and cPIM matrix and that the diffraction intensity was enhanced with the increase in the loading content of the ZIF-8 particles [28]. Furthermore, it was observed that the characteristic peak of ZIF-8 at ap-proximately 7.20 in the ZIF-8/cPIM membranes was shifted to a higher angle. However, this phenomenon was not observed in the ZIF-8/PIM-1 membranes. This suggests that the augmented rigidity of the ZIF-8 cell may be attributed to the interaction between the carboxyl group in cPIM and the Zn sites in the ZIF-8 framework [37]. The optical images shown in Figure 2b are of two casting solutions that ere left to stand for 24 h, in which b1 and b2 correspond to the ZIF-8/PIM-1 and ZIF-8/cPIM casting solutions, respectively. It was observed that some ZIF-8 particles precipitated in the ZIF-8/PIM-1 casting solution, while the ZIF-8/cPIM solution remained homogeneous. This suggests that the carboxyl groups in cPIM can promote the dispersion of ZIF-8 within the polymer, thereby enabling the preparation of uniformly dispersed high-loading MMMs [23].

The interfacial properties between the polymers and fillers were characterized using ATR-FTIR (Figure 2c and Figure S4b) analysis, which demonstrated that the characteristic peaks of Zn-N bonding at 420 cm^−1^ for ZIF-8 and C=O bonding near 1692 cm^−1^ for cPIM were observed in both MMMs. Moreover, the intensity of the Zn-N peaks was found to increase with the increase in ZIF-8 loading contents, which is consistent with the XRD results. This distinction can be observed in the red shift of the C=O peak in the cPIM, resulting from the incorporation of ZIF-8 into ZIF-8/cPIM. This indicates that the carboxyl group in the cPIM exhibits interfacial coordination with ZIF-8 [27,38]. The absence of carboxyl groups in the PIM-1 precluded the observation of a corresponding characteristic peak shift during the casting process in ZIF-8/PIM-1. These outcomes demonstrate that the cPIM matrix exhibits remarkable interfacial compatibility with ZIF-8 particles.

**Figure 3:**
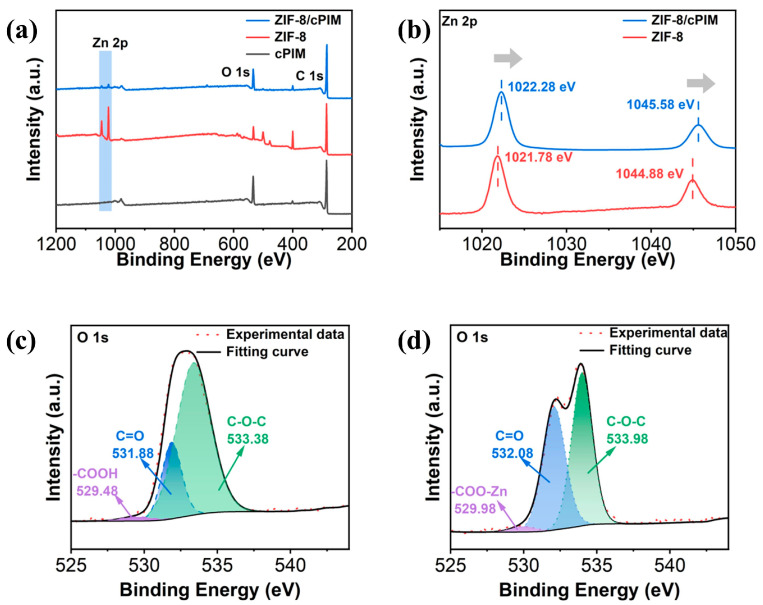
XPS spectra of ZIF-8, cPIM, and 20 wt% ZIF-8/cPIM. (**a**) Wide-scan XPS spectrum. (**b**) High-resolution XPS spectra of Zn 2p in both ZIF-8 and ZIF-8/cPIM. High-resolution XPS spectra of O 1s for cPIM (**c**) and ZIF-8/cPIM (**d**).

The interfacial coordination between fillers and polymers was further characterized by XPS. In the wide-scan XPS spectra (Figure 3a), a new Zn 2p peak appeared in the ZIF-8/cPIM membrane compared to the pristine cPIM membrane. Figure 3b displays the XPS spectra of Zn 2p in the ZIF-8 particles and ZIF-8/cPIM membrane, which shows that the binding energies of the Zn in ZIF-8 in MMM were 1022.28 and 1045.58 eV, respectively, which were shifted towards a higher binding energy compared to the ZIF-8 particles (1021.78 and 1044.88 eV). Figure 3c presents the O 1s spectra of the cPIM; the peak located at 531.88 eV is attributed to the C=O group in the imide, while the peak located at 529.48 eV is attributed to the carboxylate (–COOH) group. In the O 1s spectrum of the ZIF-8/cPIM membrane (Figure 3d), the peak of the C=O group was shifted to a higher binding energy (532.08 eV), and the carboxylate group was also shifted towards a higher binding energy (529.98 eV) to form –COO-Zn. It is evident that the carboxylate group in cPIM was interfacially coordinated with ZIF-8, which is consistent with the ATR-FTIR results. The cPIM matrix had good interfacial compatibility with ZIF-8 [28,36,38].

**Figure 4:**
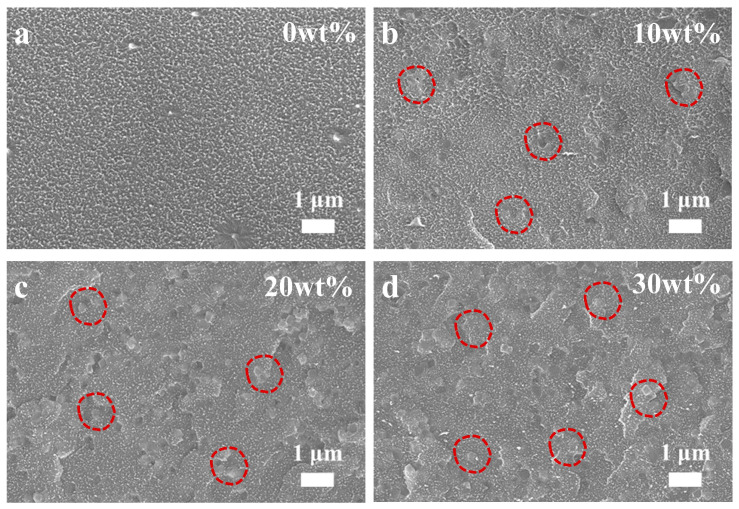
Cross-sectional SEM images of cPIM (**a**) and ZIF-8/cPIM membranes with various loadings of ZIF-8 ranging from 10 to 30 wt% (**b**–**d**).

In order to verify the superiority of cPIM in the preparation of MMMs, a series of MMMs was prepared by blending ZIF-8 with PIM-1 and cPIM, respectively. Optical images of the ZIF-8/cPIM membranes are shown in Figure S5, where it can be seen that ZIF-8 exhibited good membrane-forming properties in cPIM matrices when the loading range of ZIF-8 was 10–30 wt%; however, a film fragmentation phenomenon occurred when the loading was increased up to 40 wt%. Therefore, the subsequent discussion is based on the MMMs within the filler loading range of 10–30 wt%. Cross-sectional SEM images were used to determine the dispersion of ZIF-8 in the polymer matrix. The SEM images of the ZIF-8/cPIM MMMs show that the MOF filler was uniformly dispersed in the cPIM matrix at loadings in the range of 10–30 wt% and in an encapsulated polymer state, in which it appeared to be encapsulated by the polymer, with no agglomeration or precipitation observed (Figure 4). However, SEM images of the ZIF-8/PIM-1 membranes (Figure S6) show that ZIF-8 was well dispersed when its loading was in the range of 10–20 wt%; ZIF-8 agglomerated within the membranes when the ZIF-8 loading reached 30 wt%, resulting in the formation of non-selective defects, and was not conducive to the enhancement of gas selectivity [17,23,24,39].

### 3.2. Gas Separation Properties

**Figure 5:**
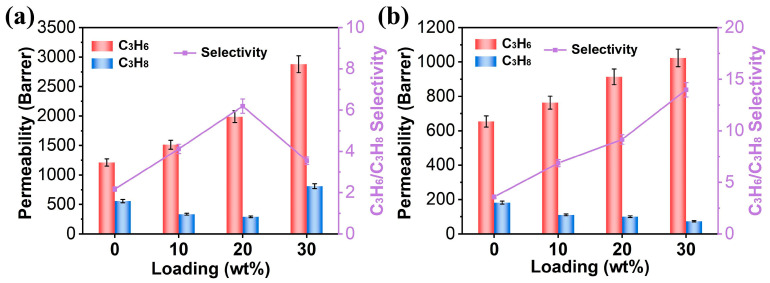
Gas separation properties of MMMs with different loading, (**a**) ZIF-8/PIM-1, (**b**) ZIF-8/cPIM, at 2 bar.

To determine the superiority of the cPIM over PIM-1 matrices, the separation performance of MMMs with 10–30 wt% ZIF-8 loadings was investigated on C_3_H_6_/C_3_H_8_ (50/50) gas mixtures (Tables S1 and S2). As shown in Figure 5a, the addition of ZIF-8 (10–20 wt%) resulted in a simultaneous increase in the C_3_H_6_ permeability and in the C_3_H_6_/C_3_H_8_ selectivity from 2 to about 6 in the ZIF-8/PIM-1 membranes. Additionally, a gradual decrease in C_3_H_8_ permeability was observed, which can be attributed to the molecular sieving effect of ZIF-8 [20,40]. However, when the loading of ZIF-8 reached up to 30 wt%, although the permeability of C_3_H_6_ continued to increase, the permeability of C_3_H_8_ also increased, resulting in a decrease in the C_3_H_6_/C_3_H_8_ selectivity to about 3.5. This is attributed to the non-selective defects caused by the aggregation of ZIF-8 within the membrane at high loading levels, which weaken the molecular sieving effect of ZIF-8. In contrast, the permeability of C_3_H_8_ continued to decrease in the case of the ZIF-8/cPIM membranes, while their C_3_H_6_ permeability and C_3_H_6_/C_3_H_8_ selectivity improved with increasing ZIF-8 loadings within the range of 10–30 wt%. In particular, when ZIF-8 was added to a 30 wt%, the C_3_H_6_ permeability was 1023 Barrer and the C_3_H_6_/C_3_H_8_ selectivity was 13.97, representing an improvement of 57% and 289%, respectively, in comparison with pristine cPIM. The excellent gas separation performance of the ZIF-8/cPIM membranes at a loading of 30 wt% is attributed to the interfacial coordination between the filler and the polymer, which enables the uniform dispersion of the filler within the polymer matrix. This allows ZIF-8 to exhibit its maximum sieving effect on C_3_H_6_/C_3_H_8_ gas separation [23,39].

**Figure 6:**
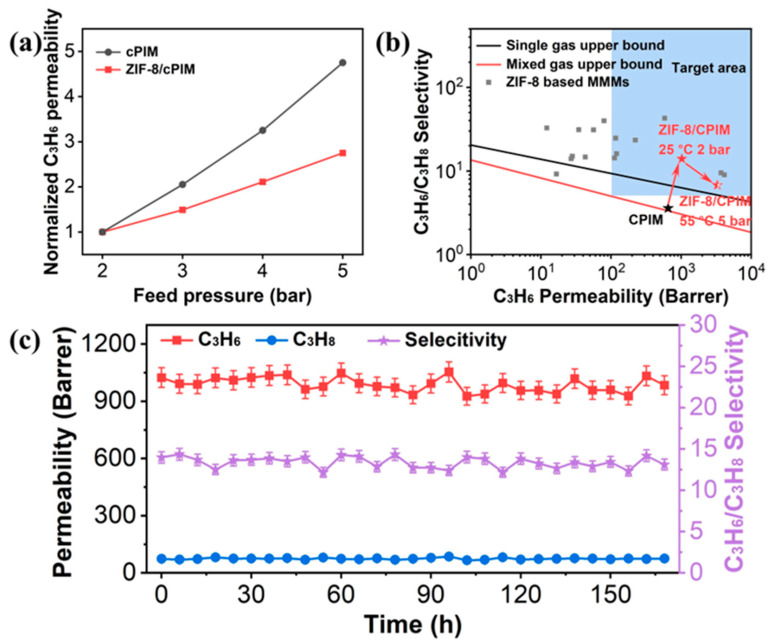
(**a**) The relationship between the C_3_H_6_ permeability and feed pressure for cPIM and ZIF-8/cPIM membranes. (**b**) Comparison of C_3_H_6_/C_3_H_8_ separation performance with other classes of membranes reported in the literature. Red star: separation performance of 30 wt% ZIF-8/cPIM membrane at 25 °C and 2 bar. Filled and half-filled red star: separation performance of 30 wt% ZIF-8/cPIM membrane at 55 °C and 5 bar. Grey squares: separation performance of ZIF-8-based MMMs reported in the literature. The inset blue area presents the desired performance for industrial C_3_H_6_/C_3_H_8_ separation. The lines are C_3_H_6_/C_3_H_8_ single-gas (black) and mixed-gas (red) upper bound, respectively [17,45]. (**c**) Long-term stability of 30 wt % ZIF-8/cPIM membrane at 2 bar and 25 °C.

During the actual operation, polymer membranes exhibit a significant pressure-induced plasticization effect. Under high-pressure conditions, the concentration of condensable gases increases, leading to polymer swelling and chain mobility, resulting in increased permeability and decreased selectivity [41,42]. The incorporation of porous MOF particles enhances the resistance to plasticization of membranes by establishing a more resilient pore architecture [18,23,25,43]. The optimal ZIF-8/cPIM membrane and the pristine cPIM membrane were tested under different transmembrane pressures, ranging from 2 to 5 bar (Figure 6a and Figure S7). The permeability of C_3_H_6_ into cPIM increased by 4.75-fold, while the permeability coefficient of C_3_H_6_ into the ZIF-8/cPIM membrane only increased by 2.75-fold. Although the C_3_H_6_/ C_3_H_8_ selectivity of both membranes decreased, the selectivity of ZIF-8/cPIM (6.57) was significantly higher than that of cPIM (1.51), demonstrating that the introduction of ZIF-8 effectively improved the anti-plasticization ability of the membrane [44].

In addition, the C_3_H_6_/C_3_H_8_ separation performance of ZIF-8/cPIM was compared with that of the most advanced ZIF-8-based MMMs [17,45]. As shown in Figure 6b, our ZIF-8/cPIM membranes were competitive with most of these membranes (Table S3), exceeding the latest upper bound. The excellent interfacial compatibility of ZIF-8-CPIM combined with the porous structure of cPIM resulted in ZIF-8/cPIM exhibiting outstanding permeability and moderate selectivity in the reported ZIF-8 MMMs. In real separation processes, the membranes must maintain high C_3_H_6_/C_3_H_8_ separation performance at higher temperature and pressure conditions. As a proof of concept, we tested our membranes’ performance using a C_3_H_6_/C_3_H_8_ (50/50) feed gas mixture under an operating temperature of 55 °C and a transmembrane pressure of 5 bar. Surprisingly, under these harsh conditions, the 30 wt% ZIF-8/cPIM membrane surpassed the current upper bound of mixed-gas separation and met the C_3_H_6_/C_3_H_8_ separation requirements for industrial membrane distillation processes as proposed by Baker et al. [25]. Furthermore, long-term testing stability is a critical benchmark for evaluating membrane performance (Figure 6c). The 30 wt% ZIF-8/cPIM membrane demonstrated outstanding stability during continuous testing for 168 h under an operating temperature of 25 °C and a transmembrane pressure of 2 bar.

**Figure 7:**
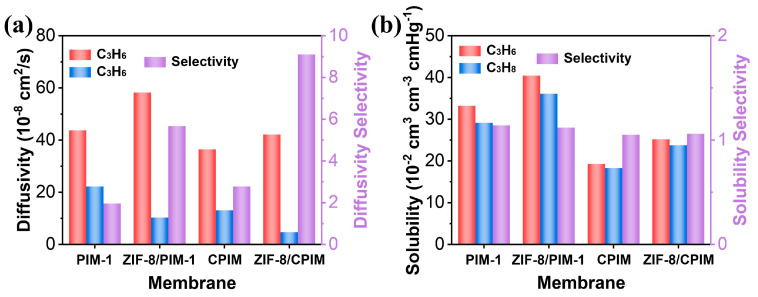
Diffusivity (**a**) and solubility coefficient (**b**) of C_3_H_6_ and C_3_H_8_ for PIM-1, cPIM, and MMMs with 20 wt% ZIF-8 loading.

In order to better understand the impact of the interfacial coordination between ZIF-8 and cPIM on C_3_H_6_/C_3_H_8_ separation, a solution–diffusion model was employed [46]. The diffusivity and solubility coefficient (Figure 7 and Table S4) of C_3_H_6_ and C_3_H_8_ for cPIM, PIM-1, and regular MMMs with 20 wt% ZIF-8 loadings were tested using a permeameter. It is evident that the incorporation of ZIF-8 led to an increase in the diffusivity and solubility of the gas to some extent. This can be attributed to the doping of the porous ZIF-8 filler, which increases the gas transport channels within the membranes and the free volume fraction of the membranes [24]. Furthermore, both MMMs exhibited a more pronounced enhancement in the selectivity of the diffusivity of C_3_H_6_/C_3_H_8_, while the selectivity of their solubility remained almost unchanged. This phenomenon was attributed to the molecular sieving effect of ZIF-8 toward C_3_H_6_/C_3_H_8_ [20]. In particular, the diffusion selectivity (9.11) and enhancement (228%) of the ZIF-8/cPIM membrane toward C_3_H_6_/C_3_H_8_ were higher than those of ZIF-8/PIM-1 (5.67 and 189%), which was explained by the good compatibility brought about by the interfacial coordination between cPIM and ZIF-8. Additionally, we compared mixed-matrix membranes (MMMs) that did not experience the ZIF-8-polymer interface compatibility enhancement, as shown in Table S5. For the ZIF-8/6FDA-DAM, ZIF-8/XLPEO, and ZIF-8/PIM-6FDA-OH membranes, the improvements in C_3_H_6_/C_3_H_8_ selectivity were 150%, 170%, and 48%, respectively, all of which are lower than the 290% improvement noted in the ZIF-8/cPIM membrane. This clearly demonstrates that the strong interface compatibility between ZIF-8 and the polymer significantly boosted its sieving performance. When ZIF-8 exhibits good compatibility with the polymer, it can reduce the non-selective pathways for C_3_H_8_ passing through the interfacial gaps, leading to a decrease in the diffusion coefficient of C_3_H_8_ and thereby enhancing the molecular sieving effect [22,39,43]. Therefore, the excellent compatibility between ZIF-8 and cPIM reduced the transport path of C_3_H_8_ across the interfacial gap, thus enhancing the sieving effect of ZIF-8 on C_3_H_6_/C_3_H_8_ within the membrane.

## 4. Conclusions

In this work, MMMs with optimal filler–polymer interfacial compatibility were developed for the efficient separation of C_3_H_6_/C_3_H_8_. A series of ZIF-8/cPIM MMMs was prepared by utilizing the coordination interaction between the carboxyl (–COOH) groups in cPIM and the Zn sites in ZIF-8. SEM images showed the uniform dispersion of ZIF-8 within the polymer matrix, while ATR-FTIR and XPS analyses indicated the creation of interface coordination between ZIF-8 and cPIM, which demonstrated outstanding filler–polymer interface compatibility. The doping of ZIF-8 resulted in the enhanced permeability of C_3_H_6_ and selectivity of C_3_H_6_/C_3_H_8_, exceeding the upper bound even at filler contents as high as 30 wt%. Solution–diffusion tests revealed that the superior enhancement in C_3_H_6_/C_3_H_8_ selectivity of the ZIF-8/cPIM membranes was mainly attributed to the improvement in the fast diffusivity of C_3_H_6_ molecules. The excellent interface compatibility between ZIF-8 and cPIM reduced the interfacial transport pathways for C_3_H_8_, thereby enhancing the sieving effect of ZIF-8 within the membrane. Furthermore, the doping of ZIF-8 improved the anti-plasticization capability of the membrane material, leading to its stability during continuous testing. Even under the harsh operating temperature of 55 °C and a transmembrane pressure of 5 bar, the membrane exhibited an outstanding C_3_H_6_/C_3_H_8_ separation performance, surpassing the current upper bound and meeting the requirements for industrial membrane applications. Therefore, this study provides a foundation for the development of novel advanced MOF-based MMMs and presents research strategies for the rational design of high-performance gas separation membranes.

## Data Availability

The original contributions presented in the study are included in the article/Supplementary Material; further inquiries can be directed to the corresponding authors.

## Supplementary Materials

The following supporting information can be downloaded at: https://www.mdpi.com/article/10.3390/polym16172545/s1, Figure S1. ATR-FTIR spectra of ZIF-8 particles; Figure S2. C_3_H_6_ and C_3_H_8_ adsorption isotherms of the measured ZIF-8 nanoparticle; Figure S3. ATR-FTIR spectra of cPIM (red) and PIM-1 (black); Figure S4. (a) XRD patterns of ZIF-8 nanoparticles and associated membranes with different filler loadings, and (b) ATR-FTIR spectra of PIM-1 and various MMMs with different filler loadings; Figure S5. Optical images of cPIM and ZIF-8 loadings of 10–40 wt% of ZIF-8/cPIM membrane (loadings in order from left to right are 0 wt%, 10 wt%, 20 wt%, 30 wt%, and 40 wt%); Figure S6. Cross-sectional SEM images of PIM-1 (a) and ZIF-8/PIM-1 membranes with varying loadings of ZIF-8 ranging from 10 to 30 wt% (b–d); Figure S7. The relationship between the C_3_H_6_/C_3_H_8_ selectivity and feed pressure for cPIM and ZIF-8/cPIM membranes. Table S1. C_3_H_6_/C_3_H_8_ mixed-gas separation performance of ZIF-8/PIM-1 membranes with different ZIF-8 loadings; Table S2. C_3_H_6_/C_3_H_8_ mixed-gas separation performance of ZIF-8/cPIM membranes with different ZIF-8 loadings; Table S3. Performance comparison of C_3_H_6_/C_3_H_8_ separation for ZIF-8/cPIM- and ZIF-8-based MMMs in the literature; Table S4. Diffusivity and solubility of C_3_H_6_ and C_3_H_8_ for PIM-1, cPIM, and MMMs with 20 wt% ZIF-8 loading at 1 bar and 25 °C. Table S5. Comparison of C_3_H_6_/C_3_H_8_ selectivity enhancement between ZIF-8/cPIM and ZIF-8 based MMMs in the literature. References [17,18,19,20,22,40,47,48,49,50,51,52,53,54,55] are cited in the Supplementary Materials.
