# Supplementary material for: Rational Matching of Metal–Organic Frameworks and Polymers in Mixed Matrix Membranes for Efficient Propylene/Propane Separation
Source: Polymers (Basel). 2024 Sep 9;16(17):2545. doi: 10.3390/polym16172545 (PMC11398130; doi:10.3390/polym16172545)
Supplement: Supplementary file 1 [file polymers-16-02545-s001.zip › polymers-3189316-supplementary.pdf]

Supporting Information

Rational matching of metal-organic frameworks and polymers in mixed matrix membranes for efficient propylene/propane separation

Zijun Yu[a, b], Yuxiu Sun[b, c,], Zhengqing Zhang[b, c], Chenxu Geng[b] *, Zhihua Qiao[b, c] *

[a.] School of Textile Science and Engineering, Tiangong University, Tianjin, 300387, China

[b.] State Key Laboratory of Separation Membranes and Membrane Processes,

[c.] School of Chemical Engineering and Technology, Tiangong University, Tianjin 300387, China.

Results and discussion

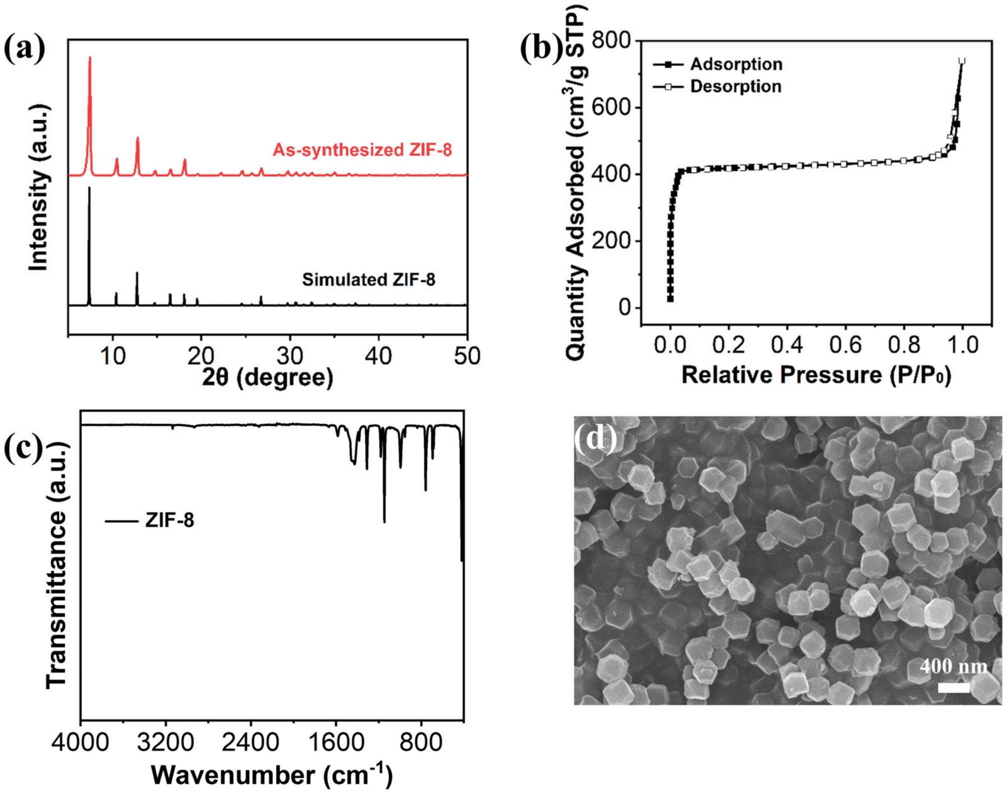

Figure S1. (a) XRD patterns of as-synthesized ZIF-8 and simulated ZIF-8. (b) The corresponding N_2 sorption isotherm of ZIF-8 at 77 K. (c) ATR-FTIR spectra of ZIF-8 particles. (d) SEM image of ZIF-8 particle sample.

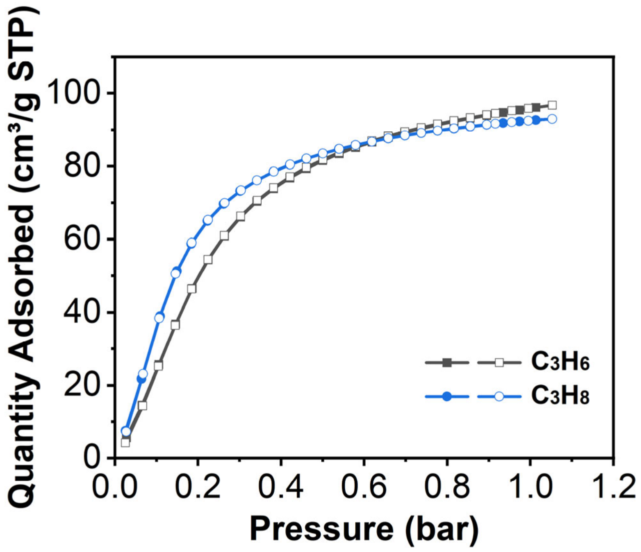

Figure S2. C_3H_6 and C_3H_8 adsorption isotherms of ZIF-8 nanoparticle measured at room temperature (298 K).

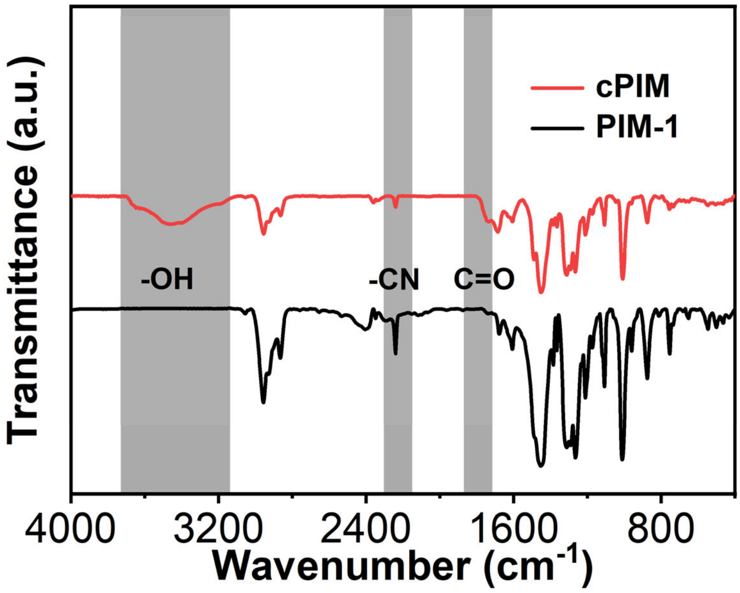

Figure S3. ATR-FTIR spectra of cPIM (red) and PIM-1 (black).

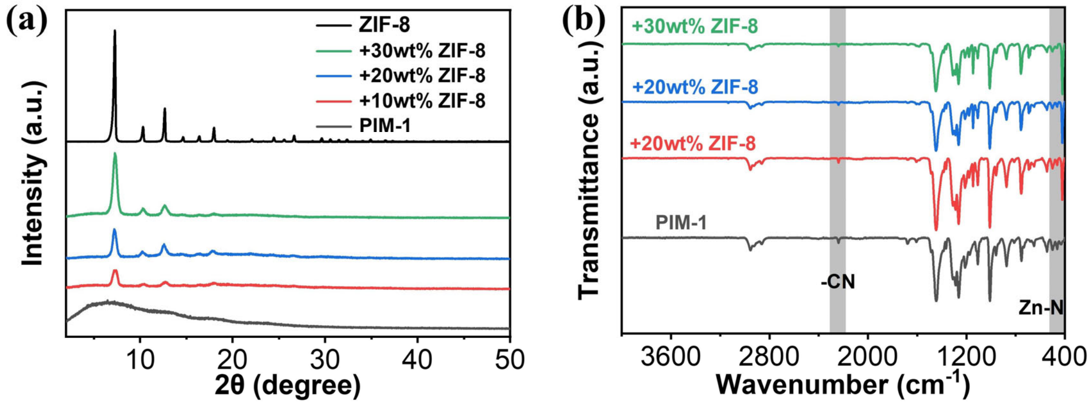

Figure S4. (a) XRD patterns of ZIF-8 nanoparticles and associated membranes with different filler loadings. (b) ATR-FTIR spectra of PIM-1 and various MMMs with different filler loadings

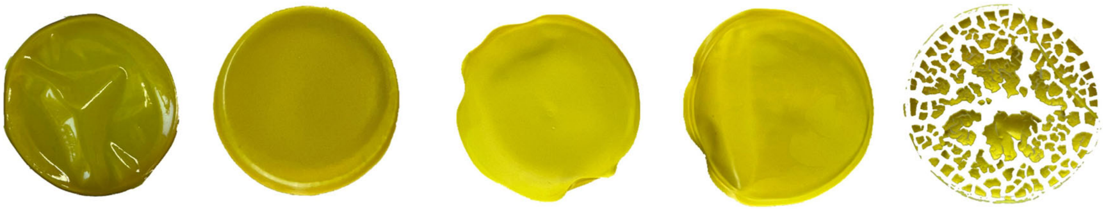

Figure S5. Optical images of cPIM and ZIF-8 loadings of 10-40 wt% of ZIF-8/cPIM membrane (The loadings in order from left to right are 0 wt% 10 wt% 20 wt% 30 wt% 40 wt%)

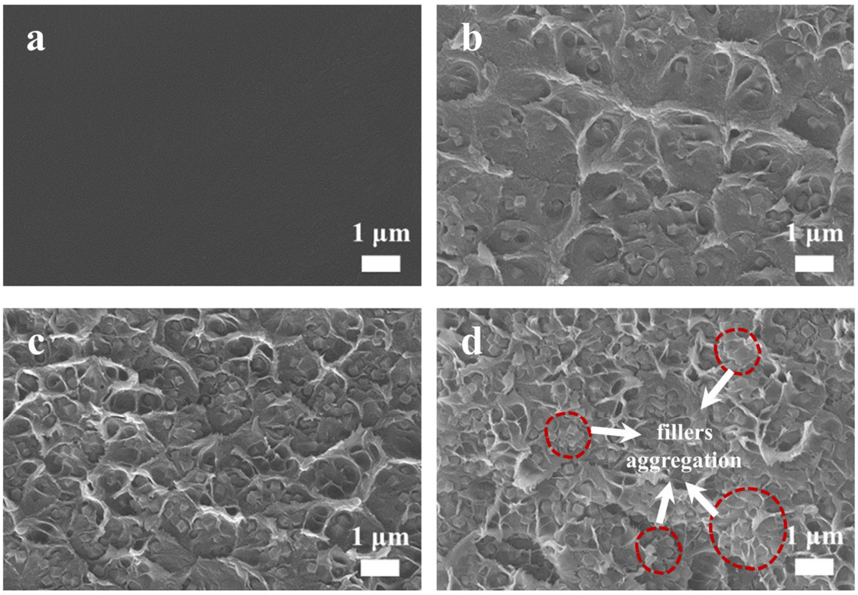

Figure S6 Cross-sectional SEM images of PIM-1 (a) and ZIF-8/PIM-1 membranes with varying loadings of ZIF-8 ranging from 10 to 30 wt% (b-d).

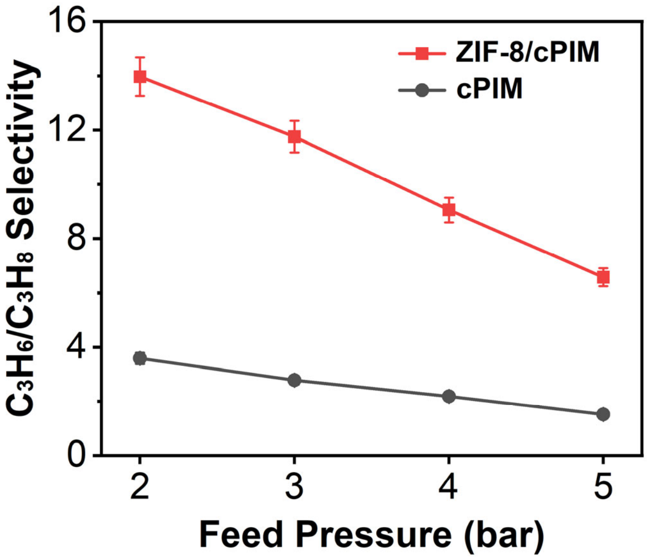

Figure S7 The relationship between the C_3H_6/C_3H_8 selectivity and feed pressure for cPIM and ZIF-8/cPIM membranes.

Table S1 C_3H_6/C_3H_8 mixed gas separation performance of ZIF-8/PIM-1 membranes with different ZIF-8 loadings.

Membrane	C_3H_6 (Barrer)	C_3H_8 (Barrer)	α
PIM-1	1211.58±60.58	558.33±27.92	2.17±0.11
+10 wt% ZIF-8	1512.55±76.63	334.63±16.73	4.12±0.23
+20 wt% ZIF-8	1988.28±99.41	288.57±14.43	6.19±0.34
+30 wt% ZIF-8	2879.22±143.96	811.04±40.55	3.55±0.18

Table S2 C_3H_6/C_3H_8 mixed gas separation performance of ZIF-8/cPIM membranes with different ZIF-8 loadings.

Membrane	C_3H_6 (Barrer)	C_3H_8 (Barrer)	α
cPIM	653.91±32.70	182.03±9.1	3.59±0.18
+10 wt% ZIF-8	763.51±38.18	111.05±5.55	6.87±0.34
+20 wt% ZIF-8	913.55±45.68	99.84±4.99	9.15±0.46
+30 wt% ZIF-8	1023.91±51.20	73.30±3.67	13.97±0.70

Table S3 Performance comparison of C_3H_6/C_3H_8 separation for ZIF-8/cPIM and ZIF-8 based MMMs in the literature

Membrane	Pressuer (bar)	C_3H_6 (Barrer)	α	Reference
ZIF-8/6FDA-DAM	2	56.2	31	[1]
ZIF-8/CNT/6FDA-Durene	2	120	16.1	[2]
ZIF-8-PIM-6FDA-OH	3.5	34.6	31.2	[3]
ZIF-8/PVAc	2	27	14	[4]
ZIF-8@DBzPBI-Bul	2.7	12.13	32.7	[5]
ZIF-8-NR/XLPEO	-	16.6	9.2	[6]
ZIF-8/XLPEO	2	28	15	[7]
$eIm_{0.2}$-ZIF-8/6FDA-DAM	1	42.94	14.7	[8]
AZIF-8/6FDA-DAM	2	79.38	39.8	[9]
Im_{52}/ZIF-8/6FDA-DAM	1	111.9	14.3	[10]
ZIF-8@Agmim/PIM-1	1	4143	9	[11]
ZIF-8-DTC/6FDA-DAM	1	582	42.8	[12]
ZIF-8 Nanoplate/6FDA-DAM	2	116.3	24.7	[13]
ZIF-8-CN@tPIM-1	1.5	222.3	23.4	[14]
ZIF-8@Ag3pz3/PIM-1	1	3708	9.5	[15]
ZIF-8/cPIM	2	1023.91	13.97	This work

Table S4 Diffusivity and solubility of C_3H_6 and C_3H_8 for PIM-1, cPIM and MMMs with 20 wt% ZIF-8 loading at 1 bar and 25 °C.

Membrane	Diffusivity (D, x 10^{-8} cm^2/s)			Solubility (S, x 10^{-2} cm^3 cm^3 $cmHg^{-1}$)		
	D_{C3H6}	D_{C3H8}	D_{C3H6}/D_{C3H8}	S_{C3H6}	S_{C3H8}	S_{C3H6}/S_{C3H8}
PIM-1	43.71	22.24	1.96	33.21	29.14	1.14
ZIF-8/PIM-1	58.25	10.27	5.67	40.45	36.12	1.12
cPIM	36.45	13.10	2.78	19.29	18.31	1.05
ZIF-8/cPIM	42.14	4.66	9.11	25.19	23.76	1.06

Table S5 Comparison of C_3H_6/C_3H_8 selectivity enhancement between ZIF-8/cPIM and ZIF-8 based MMMs in the literature.

Membrane	α (C_3H_6/C_3H_8)	Membrane	α (C_3H_6/C_3H_8)	C_3H_6/C_3H_8 selectivity enhancement (%)	Reference
6FDA-DAM	12.4	ZIF-8/6FDA-DAM	31.0	150%	1
PIM-6FDA-OH	21	ZIF-8/PIM-6FDA-OH	31.2	48%	3
XLPEO	3.4	ZIF-8/XLPEO	9.2	170%	6
cPIM	3.59	ZIF-8/cPIM	13.97	290%	This Work

Reference

[1] Zhang, C.; Dai, Y.; Johnson, J. R.; Karvan, O.; Koros, W. J. High performance ZIF-8/6FDA-DAM mixed matrix membrane for propylene/propane separations. J. Membr. Sci. **2012**, *389*, 34-42.

[2] Lin, R.; Ge, L.; Diao, H.; Rudolpha, V.; Zhu, Z. Propylene/propane selective mixed matrix membranes with grape-branched MOF/CNT filler. J. Mater. Chem. A **2016**, *4*, 6084-6090.

[3] Ma, X.; Swaidan, R. J.; Wang, Y.; Hsiung, C.; Han, Y.; Pinnau, I. Highly compatible hydroxyl-functionalized microporous polyimide-ZIF-8 mixed matrix membranes for energy efficient propylene/propane separation. ACS Appl. Nano Mater. **2018**, *1*, 3541-3547.

[4] Yu, J.; Wang, C. Q.; Xiang, L.; Xu, Y.; Pan, Y. Enhanced C_3H_6/C_3H_8 separation performance in poly(vinyl acetate) membrane blended with ZIF-8 nanocrystals. Chem. Eng. Sci. **2018**, *179*, 1-12.

[5] Kunjattu, S. H.; Ashok, V.; Bhaskar, A.; Pandare, K.; Banerjee, R.; Kharul, U. K. ZIF-8@DBzPBI-BuI composite membranes for olefin/paraffin separation. J. Membr. Sci. **2018**, *549*, 38-45.

[6] Yang, F.; Mu, H.; Wang, C. Q.; Xiang, L.; Yao, K.; Liu, L.; Yang, Y.; Han, Y.; Li, Y.; Pan, Y. Morphological map of ZIF-8 crystals with five distinctive shapes: feature of filler in mixed-matrix membranes on C_3H_6/C_3H_8 separation. Chem. Mater. **2018**, *30*, 3467-3473.

[7] Liu, D.; Xiang, L.; Chang, H.; Chen, K.; Wang, C.; Pan, Y.; Li, Y.; Jiang, Z. Rational matching between MOFs and polymers in mixed matrix membranes for propylene/propane separation. Chem. Eng. Sci. **2019**, *204*, 151-160.

[8] Park, S. H.; Jeong, H. K. In-situ linker doping as an effective means to tune zeolitic-imidazolate framework-8 (ZIF-8) fillers in mixed-matrix membranes for propylene/propane separation. J. Membr. Sci. **2020**, *596*, 117689.

[9] An, H.; Cho, K. Y.; Lyu, Q.; Chiou, D. S.; Nam, K. J.; Kang, D. Y.; Lin, L.; Lee, J. S. Facile defect engineering of zeolitic imidazolate frameworks towards enhanced C_3H_6/C_3H_8 separation performance. Adv. Funct. Mater. **2021**, *31*, 2105577.

[10] Hillman, F.; Hamid, M. R. A.; Krokidas, P.; Moncho, S.; Brothers, E. N.; Economou, I. G.; Jeong, H. K. Delayed Linker Addition (DLA) synthesis for hybrid SOD ZIFs with unsubstituted imidazolate linkers for propylene/propane and n-butane/i-butane separations. Angew. Chem. Int. Ed. **2021**, *60*, 10103-10111.

[11]Feng, X.; Peng, D.; Shan, M.; Niu, X.; Zhang, Y. Facilitated propylene transport in mixed matrix membranes containing ZIF-8@Agmim core-shell hybrid material. AIChE J. **2022**, *68*, e17707. .

[12]Song, S.; Jiang, H.; Wu, H.; Zhao, M.; Guo, Z.; Li, B.; Ren, Y.; Wang, Y.; Ye, C.; Guiver, M. D.; He, G.; Jiang, Z. Weakly pressure-dependent molecular sieving of propylene/propane mixtures through mixed matrix membrane with ZIF-8 direct-through channels. J. Membr. Sci. **2022**, *648*,

120366.

[13] Kwon, O.; Kim, M.; Choi, E.; Bae, J. H.; Yoo, S.; Won, J. C.; Kim, Y. H.; Shin, J. H.; Lee, J. S.; Kim, D. W. High-aspect ratio zeolitic imidazolate framework (ZIF) nanoplates for hydrocarbon separation membranes. Sci. Adv. **2022**, *8*, eabl6841.

[14] Wang, Z.; Wang, W.; Zeng, T.; Ma, D.; Zhang, P.; Zhao, S.; Yang, L.; Zou, X.; Zhu, G. Covalent-linking-enabled superior compatibility of ZIF-8 hybrid membrane for efficient propylene separation. *Adv. Mater.* **2022**, *34*, 2104606.

[15] Peng, D.; Feng, X.; Yang, G.; Niu, X.; Liu, Z.; Zhang, Y. In-situ growth of silver complex on ZIF-8 towards mixed matrix membranes for propylene/propane separation. *J. Membr. Sci.* **2023**, *668*, 121267.
